# Comparison of the oxidative respiratory burst and mitogen-induced leukocyte responses of camels, goats, sheep, and cows

**DOI:** 10.14202/vetworld.2022.1398-1407

**Published:** 2022-06-07

**Authors:** Abeer Al-Hamrashdi, Khalid Al-Habsi, Elshafie I. Elshafie, Eugene H. Johnson

**Affiliations:** 1 Department of Animal and Veterinary Sciences, College of Agricultural and Marine Sciences, Sultan Qaboos University, Muscat, Oman; 2 Central Veterinary Research Laboratory, Al Amarat, Khartoum, Sudan; 3 Department of Animal and Veterinary Sciences, College of Agricultural and Marine Sciences, Sultan Qaboos University, Muscat, Oman

**Keywords:** adaptive immune system, chemiluminescence, concanavalin A, innate immune system, phytohemagglutinin, pokeweed mitogen

## Abstract

**Background and Aim:**

The reports from the Ministry of Agriculture and Fisheries suggest that camels suffer less compared to goats, sheep, and cows from a number of common infectious diseases in Oman. However, there is no immunological evidence to substantiate this claim. This present study is, therefore, an attempt to study the immunological responses of camels, goats, sheep, and cows by comparing their oxidative respiratory burst of peripheral blood leukocytes (PBLs) as a marker of innate immunity occurring during phagocytosis and the mitogenic responses of their peripheral blood mononuclear leukocytes (PBMLs) as a marker of their adaptive immune response.

**Materials and Methods:**

Ten female adult animals (n = 10) were selected from each species (goats, sheep, and cows). The goats, sheep, and cows were maintained at the Agricultural Experiment Station, while camels were kept at the Royal Camel Corps (RCC). Blood samples were collected from the jugular vein in 7 mL of heparin and ethylenediaminetetraacetic acid vacutainer tubes. The oxidative respiratory burst of PBLs was measured using a chemiluminescence (CL) assay. Reactants consisted of 75 mL of whole blood diluted (1:50), 75 mL of luminol/isoluminol, and 75 mL of zymosan opsonized with non-heat inactivated serum/heat-inactivated serum or non-opsonized zymosan. CL responses were measured as relative light units and expressed as the mean count per minute and peak CL values. The mitogenic response of PBMLs to concanavalin A (Con-A), phytohemagglutinin (PHA), and pokeweed mitogen (PWM) was tested using a WST-8 assay and read spectrophotometrically at 450 nm.

**Results:**

The present findings showed that camel PBLs generate significantly higher CL responses, both intracellularly as well as extracellularly, with zymosan opsonized with autologous serum. Camel PBLs demonstrated a significantly higher (p = 0.001) response when stimulated with zymosan opsonized with heat-inactivated serum compared to those of goat, sheep, and cow lymphocytes from camels exhibited significantly higher (p = 0.001) stimulation indices (SI) with Con-A, PHA, and PWM.

**Conclusion:**

The present study suggests that camels are capable of mounting both superior innate as well as adaptive immune responses and provide immunological evidence supporting the belief of some authors, who have proposed that camels are less susceptible to a number of common infectious diseases than other domesticated ruminants.

## Introduction

It has been hypothesized by several authors that camels are more resistant to a number of infectious diseases in comparison to other ruminants [1, 2], and it has been suggested that this might be an evolutionary adaptation of camels to be able to survive under harsh environmental conditions.

Larska *et al*. [3] reported that camels are more resistant to foot and mouth disease (FMD) compared to the sheep, maintained in the same environment, and are less susceptible to tetanus and bovine spongiform encephalopathy [4]. Clinical manifestations are also more pronounced among other farm animals with rift valley fever than camels [5]. Despite these observations, no definitive immunological studies could provide evidence that camels differ in their adaptive or specific immunological responses.

In recent years, camelids have been shown to have antibodies that differ structurally from those of other mammalian species. Mammalian antibodies are generally described as heterotetrameric antibodies containing two heavy and two light chains [6]. However, Hamers-Casterman *et al*. [7] identified unique structural antibodies of camels consisting of only heavy chains. These antibodies have recently been described as nanobodies [8] or heavy chain antibodies [9]. This is because they do not have the typical CH1 domain that functions to anchor light chains [9]. The lack of CH1 also results in these antibodies having smaller heavy chains than other mammalian species [8]. Because of their minuteness, they can penetrate and recognize small antigenic sites that conventional antibodies cannot recognize [10, 11].

However, there is a scarcity of information available regarding the innate immune response of camels. Johnson *et al*. [12] presented preliminary findings, which demonstrated that neutrophils isolated from the peripheral blood of camels exhibited a higher oxidative respiratory burst during the phagocytic process than those of neutrophils from sheep. The oxidative respiratory burst is the most effective weapon of neutrophils and monocytes/macrophages of the innate immune system [9] against fungal and bacterial pathogens [13–16]. Early in the phagocytic process, these cells generate reactive oxygen species (ROS) to combat invading microorganisms such as superoxide radical (O_2_), hydrogen peroxide (H_2_O_2_), hypochlorous acid (HOCl), and hydroxyl radical (OH). These ROS emit photons that can be measured as chemiluminescence (CL). The resultant CL can be enhanced by the presence of amplifiers such as luminol [17–19] which measures intracellular and extracellular ROS [20] or isoluminol which detects ROS generated extracellularly [21]. The detection of ROS emitted using CL assays is a well-documented means of semi-quantifying phagocytic activity [17–19].

The present study was designed to compare the CL responses of leukocytes in the whole blood of camels, goats, sheep, and cows to determine if they potentially have a superior innate immune response. Similarly, we compared the mitogenic response of peripheral blood lymphocytes to determine if camels differ in their adaptive immune response.

## Materials and Methods

### Ethical approval

The project adhered to the ethical code of Sultan Qaboos University, Oman, which serves as the guide for conducting the experiments. Blood samples were collected by experienced technical staff from Sultan Qaboos University or the Royal Camel Corps (RCC).

### Study period and location

The study was conducted from September 2019 to March 2020 on sheep, goats, cows, and camels housed in Agricultural experiment Station AES and Royal Camel Corps (RCC) located in the Muscat area in the north of mainland Oman.

### Animals and sampling

Ten apparently healthy female adult sheep, goats, and cows from the Agricultural Experiment Station (AES) at Sultan Qaboos University and camels (n = 10) from the RCC were randomly selected for this study. Only females were used because of the limited number of males available, as they are kept solely for the purpose of breeding. For 6 months, the animals were housed in large open enclosures at the AES and RCC.

Blood samples (7 mL) were collected by jugular venipuncture into ethylenediaminetetraacetic acid, heparinized, and plain Vacutainer tubes (EUROMED International, European Union) for hematological evaluation, CL and mitogenesis assays, and opsonization of zymosan, respectively.

### Isolation of mononuclear leukocytes and cell viability counts

Mononuclear leukocytes from sheep, goats, cows, and camels were isolated by differential density centrifugation as previously described by Johnson *et al*. [4] with a minor modification of the protocol relative to the centrifugation time and speed used for camel samples. Briefly, 6 mL of camel heparinized blood was layered over 3 mL of Ficoll-Histopaque^®^- 1077 solution (Sigma Chemicals Co., St Louis, Missouri, USA) in conical siliconized 15 mL centrifuge tubes (Kartell S.p.A Noviglio, Italy and CellStar^®^, Germany) to isolate enriched cell preparations of mononuclear leukocytes by differential density centrifugation as described by Johnson *et al*. [22]. The tubes were centrifuged at 1000× *g* for 20 min at 21°C. The mononuclear layer was isolated in conical siliconized 15 mL centrifuge tubes and suspended in 10 mL of phosphate-buffered saline (PBS) (pH ~ 7.4). The suspension was centrifuged at 600× *g* for 5 min at 21°C. The cells containing pellets were washed three times and suspended at 200× *g* for 10 min. Finally, the pellet was re-suspended in 1 mL of RPMI-1640 (Sigma Chemicals Co.) containing 10% of fetal bovine albumin (Sigma Chemicals Co.) and 200 mM L-glutamine, 10,000 units of Penicillin, and 10 mg/mL of streptomycin (Sigma Chemicals Co.).

Cell viability counts were conducted, as previously described by Johnson *et al*. [23]. Only samples with ≥ 95% viability were employed in the study. Final counts were adjusted to 1 × 10^6^ cells/mL.

### Mitogenic assay

Three mitogens (Sigma Chemicals Co.) were employed in the present study, namely phytohemagglutinin (PHA), pokeweed mitogen (PWM), and concanavalin A (Con-A), as previously described by Johnson *et al*. [24]. In a series of pilot studies, the optimal end concentration for the four species for each mitogen used was 10 μg/mL.

The mitogenic responses were measured in 96-well microtiter plates in which 100 μL of cell suspension and 15 μL of mitogen were mixed in each well. Cells were incubated at 37°C for 72 h in a humidified 5% CO_2_ incubator. Thereafter, 10 μL of WST-8 (highly water-soluble tetrazolium salt) (Sigma Chemicals Co.) was added. After 4 h of incubation, the optical density of each well was measured spectrophotometrically at 450 nm in a multiskan spectrum machine (Thermo Electron Corporation, Vantaa, Finland). The results were recorded as the mean of optical density (MOD) and stimulation index (SI). Each sample was tested in triplicate.

To rule out experimental bias and random errors, samples were tested in triplicate and results were recorded as the MOD and stimulation index (SI).


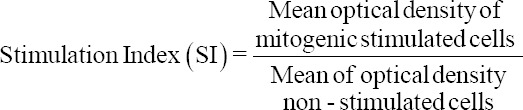


### Preparation of luminophores and opsonization of zymosan

Two luminophores (luminol and isoluminol) and one stimulant (zymosan) (Sigma Chemicals Co.) were prepared as previously described by Pavelkova and Kubala [25] at 20 and 40 mmol/L, respectively. While both luminophores were made freshly on the same day of each new experiment, the stimulant was aliquoted and stored at −20°C for subsequent CL assays.

The opsonization of zymosan was described by Johnson *et al*. [23]. Briefly, 100 mg of zymosan particles (Sigma Chemicals Co.) were added to 2 mL of PBS in 15 mL conical tubes (Kartell S.p.A and CellStar tubes, Germany). The mixture was heated in a boiling water bath for 1 h with frequent mixing. Consequently, 10 mL of PBS was added to the tubes and the mixture was centrifuged at 1900× *g* for 15 min, and then pellets were washed three times with PBS. Finally, the pellets were re-suspended in 2 mL of PBS to give a final concentration of 50 mg/mL.

Before the assay, 5 mg/0.1 mL of non-opsonized zymosan A was added to 0.9 mL of homologous serum (from each animal species) and incubated at 37°C with frequent mixing for 15 min. The opsonized zymosan was suspended in 10 mL of RPMI-1640 (Sigma Chemicals Co.) and centrifuged at 850× *g* for 20 min. The procedure was repeated two times. The supernatants were discarded, and the pellet was re-suspended in 4 mL of RPMI-1640 (Sigma Chemicals Co.). Opsonization was done in each case with pooled autologous serum from ten animals as previously described by Johnson *et al*. [23].

### Zymosan opsonized with a heat-inactivated serum of camel peripheral blood leukocytes (PBL)

The zymosan was treated in the same fashion with pooled sera except in this experiment; the sera were heated 56°C for 30 min before being employed for opsonization. A preliminary study showed that no significant response was observed in goats, sheep, and cows PBL when zymosan opsonized with heat-inactivated serum (unpublished data were performed by Prof. Eugene H. Johnson at Sultan Qaboos University).

### CL assay

The procedure for CL assay was described by Neamah *et al*. [26] with minor modification in regard to the amount of luminophores and stimulants as well as using the whole blood in the following manner. Briefly, 96-well microtiter plates were used for the CL assay. Reactants in each well consisted of 75 μL of whole blood diluted (1:50) in RPMI-1640 (Sigma, Chemicals Co.) and 75 μL of luminol/isoluminol (Sigma Chemicals Co.). After 10 min of incubation at 37°C, 75 μL of opsonized zymosan was added to initiate the CL reaction. Photon emission was measured in an Ascent Luminometer (Thermo Electron Corporation) at 37°C with intermittent shaking every 2 min for 1 h to generate the kinetic curves. CL results were reported as arbitrarily relative light units, and the results were calculated as average counts per minute (CPM) as well as average peak values. Each sample was tested in triplicate.

### Statistical analysis

All data generated from this research were analyzed using JMP statistical package (JMP^®^, version 9. SAS Institute Inc., Cary, NC, USA, 1989–2021). The continuous parameters of CL (CPM) were entered into a mixed model analysis of variance containing animal groups, time, and the interaction of groups per time as the main effects. The animal was considered as a random factor in the model. The contrast was made when the main effect was found to be significant.

The continuous parameters of mitogenesis such as Con-A, PHA, and PWM were entered into a one-way analysis of variance (ANOVA) based on the four animal groups under investigation. A further analysis using Tukey’s test was applied to evaluate the differences between the four groups revealed by ANOVA.

p = 0.05 and 95% confidence interval were used to declare statistical significance for CL and mitogenesis results which were presented as least-square means and means ± standard deviation of the different animal groups (goat, sheep, cow, and camel) for the CL and mitogenesis results, respectively.

## Results

### CL assay

#### Luminol-dependent CL

The mean CPM of camel PBL (3390.37 ± 37.57) was significantly higher (p = 0.001) than goats (2176.04 ± 37.66), sheep (1267.47 ± 37.70), and cows (1391.11 ± 37.70). There was a significant difference across the four animal groups, F = 670.72, p = 0.001, and a significant difference between time F = 48.30, p = 0.001. There was also a statistically significant interaction between group and time, F = 7.58, p = 0.001. The mean CPM of goats was significantly different (p = 0.001) than sheep and cows. However, there was no statistical difference (p > 0.05) in the CPM between sheep and cows (Table-1).

**Table 1:** Comparison of the luminol-dependent mean CPM and peak CL value of camels, goats, sheep, and cows.

Species	Mean CPM ± SD	Peak CL value ± SD
Camels	3390.37 ± 37.57*	4748.88 ± 228.29*
Goats	2176.04 ± 37.66**	2967.5 ± 194.8**
Sheep	1267.47 ± 37.70	1687.13 ± 185.71
Cows	1391.11 ± 37.70	2250.77 ± 244.22

* Both mean CPM and peak CL values for camels are significantly different from that of the other three groups.

** Both mean CPM and peak CL values for goats are significantly different from that of sheep and cows.

CPM=Counts per minute, CL=Chemiluminescence,

SD=Standard deviation

**Figure-1:**
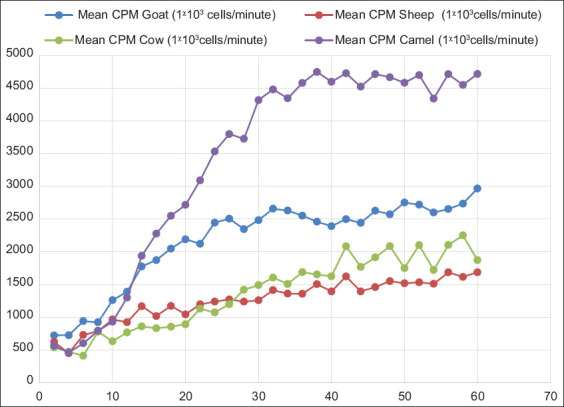
Comparison of luminol-dependent chemiluminescence of peripheral blood leukocytes from camels, goats, sheep, and cows.

The kinetic response of luminol-dependent CL was similar among the species. A significant interaction effect (p < 0.001) of luminol-dependent CL between group and time during the experiment was observed. After 18 min, the CL response of camel PBL became significantly higher than the other three groups and reached its peak (4748.88 ± 228.29) at 38 min. The CL value of goats at 18 min was significantly higher than those of sheep and cows and reached its peak value (2967.5 ± 194.80) at 60 min, as shown in Table-1. The peak values of sheep and cows were not significantly different (Figure-1).

#### Isoluminol-dependent CL

The mean CPM of camel PBL (2501.95 ± 34.50) was significantly higher (p = 0.001) than that of goats (1578.37 ± 34.47), sheep (1051.50 ± 34.58), and cows (1178.14 ± 34.87). In addition, there was a significant difference between the four animal groups, F = 335.126, p = 0.001, and a significant difference between time F = 33.1203, p = 0.001. There was also the statistical significance of group/time interaction, F = 3.4802, p = 0.001. The mean CPM of goats was significantly higher (p = 0.001) than sheep and cows. However, there was no significant difference (p > 0.05) of CPM between sheep and cows (Table-2).

**Table 2:** Comparison of the isoluminol-dependent mean CPM and peak CL values of camels, goats, sheep, and cows.

Species	Mean CPM ± SD	CPM Peak ± SD
Camels	2501.95 ± 34.50*	3554.1 ± 279.44*
Goats	1578.37 ± 34.47**	2346.88 ± 130.37**
Sheep	1051.50 ± 34.58	1627.23 ± 150.28
Cows	1178.14 ± 34.87	1756.29 ± 189.13

* Both mean CPM and peak CL values for camels are significantly different from that of the other three groups.

** Both mean CPM and peak CL values for goats are significantly different from that of sheep and cows.

CPM=Counts per minute, CL=Chemiluminescence,

SD=Standard deviation

**Figure-2:**
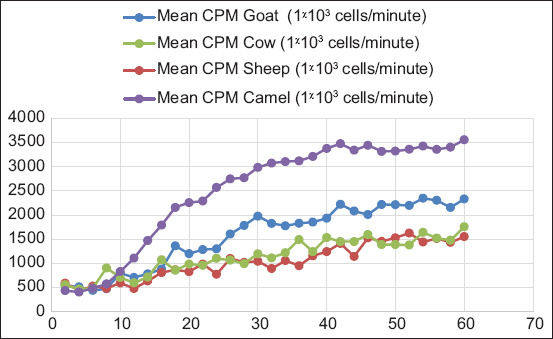
Comparison of isoluminol-dependent chemiluminescence of peripheral blood leukocytes from camels, goats, sheep, and cows.

The kinetic response of isoluminol-dependent CL was similar among the four species. There was a significant interaction effect (p < 0.001) of isoluminol-dependent CL among group/time during the experiment. After 18 min, the CL response of camel PBL became significantly different from that of the other three groups and reached its peak (3554.1 ± 279.44) at 60 min. The CL value of goats at 26 min was significantly higher than sheep and cows and reached its peak value (2346.88 ± 130.37) at 54 min, as shown in Figure-2. The peak values of sheep and cows were not significantly different.

### CL of camel PBL with zymosan opsonized with heat-inactivated serum

**Figure-3:**
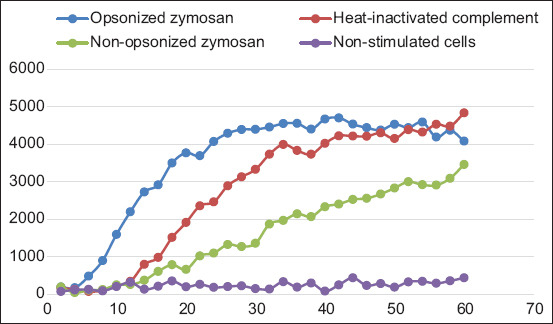
Comparison of luminol-dependent chemilumine scence responses of camel peripheral blood leukocytes with opsonized zymosan, zymosan opsonized with heat-inactivated serum, non-opsonized zymosan, and non-stimulated cells.

The CL response of camel PBL with zymosan opsonized with non-heat inactivated serum expressed in CPM (3566.83 ± 79.90) was significantly higher (p = 0.001) than zymosan treated with heat-inactivated serum (2816.72 ± 79.90), non-opsonized zymosan (1622.25 ± 81.94), and non-stimulated cells (251.47 ± 85.28), as shown in Table-3. The CPM of zymosan opsonized with heat-inactivated serum was significantly higher than that of non-opsonized zymosan and non-stimulated cells. Statistical difference was also observed between the non-opsonized zymosan and the non-stimulated cells, as shown in Figure-3. In contrast, in a series of previously performed experiments, PBL from goats, sheep, and cows showed that the CL responses produced with zymosan opsonized with heat-inactivated sera were not significantly different from non-opsonized zymosan (unpublished data).

**Table 3:** Comparison of luminol-dependent CL response of camel PBL with opsonized zymosan, zymosan opsonized with heat-inactivated serum, non-opsonized zymosan, and non-stimulated cells.

Protocol	Mean counts per minute ± standard deviation
Opsonized zymosan	3566.83 ± 79.90*
Zymosan opsonized with heat-inactivated serum	2816.72 ± 79.90**
Non-opsonized zymosan	1622.25 ± 81.94***
Non-stimulated cells	251.47 ± 85.28

* The CL response of camel PBL with opsonized zymosan is significantly higher among the others.

** The CL response of camel PBL with opsonized zymosan with heat-inactivated serum is significantly higher among the non-opsonized zymosan and non-stimulated cells.

*** The CL response of camel PBL with non-opsonized zymosan is significantly higher than non-stimulated cells.

PBL=Peripheral blood leukocytes, CL=Chemiluminescence

### Mitogenesis

#### Con-A

**Figure-4:**
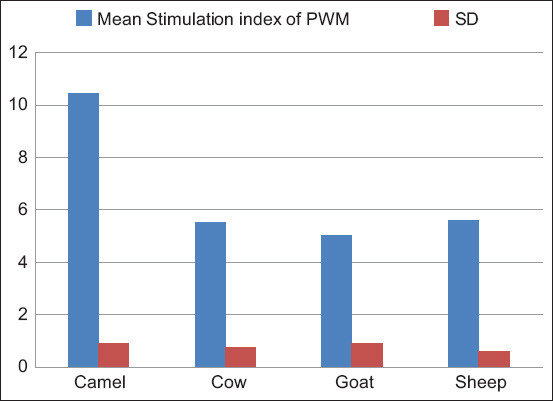
Comparison of stimulation indices of peripheral blood mononuclear leukocytes from camels, goats, sheep, and cows with concanavalin A.

One-way independent ANOVA yielded a significant difference between the four animal groups, F (3, 116) = 233.96, p < 0.05. The Tukey’s test showed that the mean stimulation index of camel peripheral blood mononuclear leukocytes (PBML) (16.57 ± 1.87) was significantly higher (p < 0.05) than those of goat (8.33 ± 0.97), sheep (7.09 ± 2.04), and cows (7.40 ± 1.37). In addition, the mean stimulation index of goat PBML was significantly higher (p < 0.05) than that of sheep. However, the mean stimulation indices (SI) of sheep and cow PBML were not incomparably different (p > 0.05), as shown in Figure-4 and Table-4.

**Table 4:** Comparison of stimulation indices of peripheral blood mononuclear leukocytes of camels, goats, sheep, and cows stimulated with Con-A, PHA, and PWM.

Variables	Mitogenesis (mean of stimulation indices ± Standard deviation)				F-test	DF	p-value

	Goat	Sheep	Cows	Camel			
Con-A	8.33 ± 0.97	7.09 ± 2.04	7.40 ± 1.37	16.57 ± 1.87	233.96	3	0.001
PHA	3.61 ± 0.58	3.77 ± 0.61	3.90 ± 0.76	6.95 ± 0.58	188.66	3	0.001
PWM	5.03 ± 0.90	5.62 ± 0.62	5.53 ± 0.78	10.43 ± 0.92	288.78	3	0.001

Con-A=Concanavalin A, PHA=Phytohemagglutinin, PWM=Pokeweed mitogen

#### PHA

**Figure-5:**
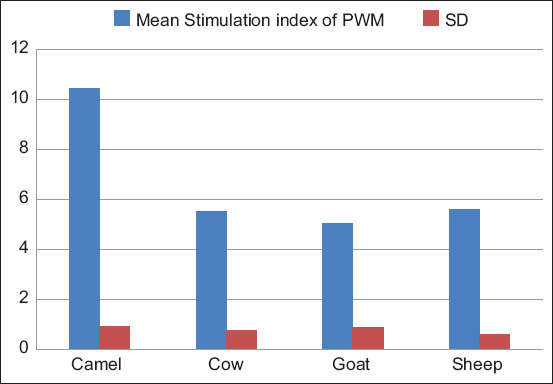
Comparison of stimulation indices of peripheral blood mononuclear leukocytes from camels, goats, sheep, and cows with phytohemagglutinin.

One-way ANOVA yielded a significant difference between animal groups, F (3, 116) = 188.66, p < 0.05. The Tukey’s test showed that the mean stimulation index of camel PBML (6.95 ± 0.58) was significantly higher (p < 0.05) than those of goat (3.61 ± 0.58), sheep (3.77 ± 0.61), and cows (3.90 ± 0.76). However, the mean SIs of goat, sheep, and cow PBML were not significantly different (p > 0.05), as observed in Figure-5 and Table-4.

#### PWM

**Figure-6:**
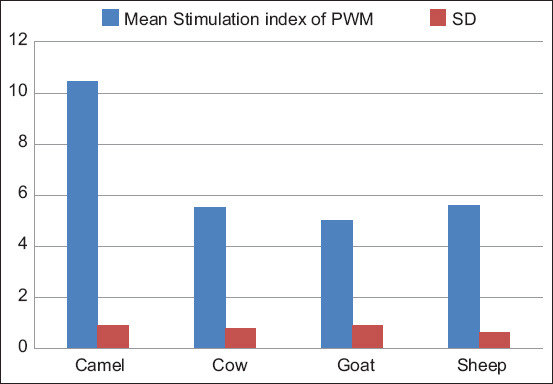
Comparison of stimulation indices of peripheral blood mononuclear leukocytes from camels, goats, sheep, and cows with pokeweed mitogen.

One-way ANOVA yielded a significant difference between animal groups, F (3, 116) = 288.78, p < 0.05. The Tukey’s test showed that the mean stimulation index of camel PBML (10.43 ± 0.92) was significantly higher (p < 0.05) than that of goat (5.03 ± 0.90), sheep (5.62 ± 0.62), and cows (5.53 ± 0.78). However, the mean stimulation index of goats, sheep, and cow PBML was not significantly different (p > 0.05) as noted in Figure-6 and Table-4.

**Figure-7:**
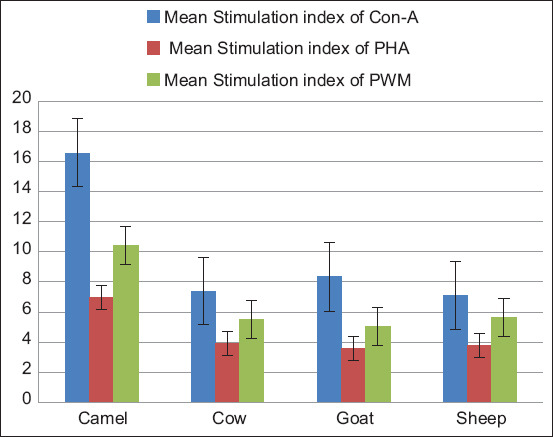
Comparison stimulation indices of peripheral blood mononuclear leukocyte of camels, goats, sheep, and cows stimulated with concanavalin A, phytohemagglutinin and pokeweed mitogen.

A comparison of the spectrophotometric mitogenic responses of PBML of all the experiment animal species stimulated with the three mitogens were further indicated in Figure-7.

## Discussion

Several authors have hypothesized that camels are more resistant to a number of infectious diseases in comparison to other ruminants [1, 2]. It is well established that camels produce unique antibodies, consisting primarily of heavy chains [27–30]. However, there is only a scarcity of information pertaining to the innate immune response of this animal species.

As a result of several preliminary studies, we hypothesized that camel’s exhibit a superior phagocytic response. We, therefore, opted to compare the CL responses generated by the PBL of camels with those of sheep, goats, and cows.

To circumvent the issue as to whether PBL might respond differently when isolated from the milieu of whole blood, we performed CL assays using a whole blood technique. As a phagocytic target, we employed the use of zymosan A with opsonized homologous sera from each of the four tested animal species.

### CL responses

#### Luminol-dependent CL

The average CPM of camel PBL with homologous opsonized zymosan A (3390.37 ± 37.57) was significantly higher (p = 0.001) than those of goats (2176.04 ± 37.66), sheep (1267.47 ± 37.70), and cows (1391.11 ± 37.70) (Table-1), and the average CPM of goat PBL was significantly higher than those of sheep and cows. Johnson *et al*. [12] preliminary findings demonstrated that camel neutrophils exhibited a higher CL response than sheep. In contrast to our findings, Cooray *et al*. [31] described no significant difference in the kinetic luminol-dependent CL response of camel neutrophils isolated from blood compared to those of humans and cows [32, 33].

However, there is a methodological difference between the present study and that of Cooray *et al*. [31]. Whereas the current work studied luminol-dependent CL from whole blood, Cooray *et al*. [31] used isolated neutrophils. Several authors have demonstrated that monocytes also contributed to the CL response. However, as they constituted < 2% of the PBL it is unlikely that they contribute significantly to the generation of ROS. In agreement with this, Peterson *et al*. [34] observed that the phagocytic ability of human neutrophils was two times higher than those of human monocytes against *Staphylococcus aureus, Escherichia coli*, and *Listeria monocytogenes*. Nelson *et al*. [35] noted that a third of the CL was generated from human monocytes when stimulated by *S. aureus*, *Candida albicans*, and opsonized zymosan.

Differences in CL responses among mammalian species have been demonstrated in the findings of Young and Beswick [36]. They observed that oxygen consumption of human neutrophils was higher than those of sheep, cows, and pigs and determined that the rate of superoxide production was higher in human neutrophils. They concluded that the neutrophils from domesticated animals consume less oxygen and produce less superoxide than those from humans in the presence of homologous opsonized zymosan. Similarly, extrapolating from this finding, it can be postulated that camel PBL likely also consumes more O_2_ and generates more ROS than PBL from goats, sheep, and cows.

Other possible reasons for the superior generation of ROS by camels may be that they generate more complement components against zymosan (C3b and iC3b) or bind more potent to their corresponding receptors. This had been described by Lieberman *et al*. [37], who observed that there was an increase in CR3 ligands on the surface of activated human neutrophils when stimulated with opsonized zymosan and thus may be a partial explanation as to why human neutrophils have been reported to generate more CL than neutrophils of sheep, cows, and pigs [36].

#### Isoluminol-dependent CL

It was of interest to study isoluminol-dependent CL. Isoluminol is unable to penetrate lipid-rich cell membranes and therefore, CL amplified with isoluminol represents the generation of ROS outside the cell [20, 38–40], in contrast to luminol that amplifies CL both inside and outside of cells [20, 41]. The average CPM of camel PBL with homologous opsonized zymosan A (2501.95 ± 34.50) was significantly higher (p = 0.001) than those of goats (1578.37 ± 34.47), sheep (1051.50 ± 34.58), and cows (1178.14 ± 34.87). The present findings were, therefore, similar to those of JančInová *et al*. [21] and Pavelkova and Kubala [25], who demonstrated that luminol generated higher levels of CL than isoluminol amplified CL in humans and rats PBL, respectively.

**Figure-8:**
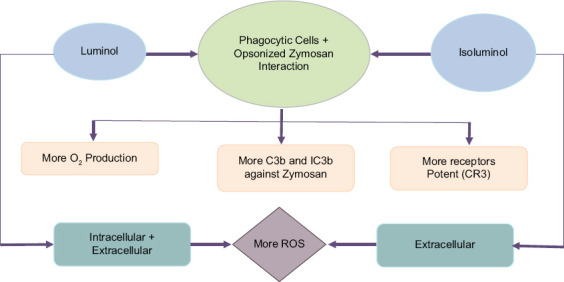
Schematic diagram of the reactive oxygen species production by camels’ phagocytic cells.

JančInová *et al*. [21] reported that the amino group of isoluminol is further from oxygen and results in isoluminol being more hydrophilic than luminol [39]. Consequently, the movement of isoluminol through the membranes of PBL is restricted [22, 39, 42] and produces a lower CL response than that amplified with luminol [25], as indicated in the schematic diagram (Figure-8).

### CL of camel PBL with zymosan opsonized with heat-inactivated serum

The data from the present study demonstrated that camel PBL generated a higher CL response with opsonized zymosan than with non-opsonized zymosan and interestingly, PBL from camels generated a significantly higher CL response in the absence of opsonins when compared to PBL from goats, sheep, and cows (unpublished observation). Small *et al*. [43] observed that the CL response of peripheral blood mononuclear cells (PMN) was higher with opsonized zymosan than with non-opsonized zymosan. They attributed this primarily to ic3b, and to a lesser degree to C3b and IgG coated opsonized zymosan. Furthermore, they found that in the absence of opsonins, glucan polysaccharide components on the surface of the zymosan interacted with CR3 on the membrane of human phagocytic cells, primary neutrophils, but to a much lesser extent monocytes and eosinophils and triggered a CL response in human whole blood. Moreover, Lieberman *et al*. [37] showed that the stimulation of opsonized zymosan upregulated arachidonic acid metabolism, which is very important in NADPH activity.

It is not inconceivable that heat-stable opsonins of camel components, such as natural antibodies against the zymosan are present in serum. In addition, natural occurring IgM antibodies against zymosan in mice have been demonstrated by Lobo *et al*. [44]. The natural antibodies are primarily of the IgM isotypes and exist against *Pneumocystis* [45–47], fungi containing glucan as found on zymosan [44], and influenza virus activating the complement and mediate the neutralization [48].

### Mitogenesis

Specific plant lectins are known to be potent mitogens. However, the present study employed lectins that stimulate T cells (Con-A and PHA) [49] and both T and B cells (PWM) [50].

The present study showed that the mean stimulation index of camel PBML stimulated with Con-A (16.57 ± 1.87) was significantly higher (p < 0.05) than those of goats (8.33 ± 0.97), sheep (7.09 ± 2.04), and cows (7.40 ± 1.37), as shown in Table-4. Furthermore, as observed with CL among the four species, goat PBML exhibited a statistically significant higher mitogenic response with Con-A than those from sheep.

Some studies have shown that although Con-A is a T-cell mitogen, under certain conditions, T-cell activation by Con-A also results in a secondary B-cell activation and clonal expansion [51, 52]. Therefore, it can be postulated that antigen stimulation of camel T-cells might result in a higher antibody response. Further studies should be conducted to test this hypothesis of camel T-cells.

The mean stimulation index of camel PBML (10.43 ± 0.92) was significantly higher (p < 0.05) than those of goats (5.03 ± 0.90), sheep (5.62 ± 0.62), and cows (5.53 ± 0.78), in the presence of PWM. Although it cannot be determined from the present study whether a specific subpopulation of lymphocytes from camels is stimulated by PWM, it has been demonstrated in cows [53], humans [54], and mice [55] that PWM is capable of stimulating both T and B cells. Indeed, when T-cells were removed from lymphocytes by treating the cells with anti-CD4 antibody and depleted by a fluorescent activated cell sorter, there was a lowering of PWM-induced activity [53]. Other studies have demonstrated that the number of T-cells presented in the culture stimulated with PWM increased the number of intracytoplasmic immunoglobulins in murine and human B-lymphocytes [54, 55].

The third mitogen tested in the current study was PHA. The mean stimulation index of camel PBML with PHA (6.95 ± 0.58) was significantly higher (p < 0.05) than those of goats (3.61 ± 0.58) sheep (3.77 ± 0.61), and cows (3.90 ± 0.76).

However, there are differences of opinion as to whether PHA stimulates B cells. Some authors suggest that PHA activates CD8+ suppressor T-cells and, consequently, suppressor B-cell differentiation [56, 57]. Contrary to this finding, Lin *et al*. [58] provided evidence that PHA can induce mitosis of B-cells. However, the mitosis of B-cells occurred after 6 days in cultures and it has been argued that the proliferation of B-cells was due to the fetal calf serum found in the culture, as this also occurred in unstimulated cells [59]. Lin *et al*. [58] suggested that the activation of B-cells might be due to the co-stimulation of B-cells by both PHA and fetal calf serum. Furthermore, it has been postulated that PHA might lead to B-cell proliferation but not to further differentiation. In contrast, PWM is capable of inducing both B-cells proliferation as well as differentiation [57].

## Conclusion

In total, these findings provided evidence that camels demonstrated superior innate and adaptive immune responses and gave credibility to the widespread belief that camels are likely capable of generating a more potent immune response to a number of bacterial and viral pathogens and thus are less susceptible to certain diseases, commonly, found in other domesticated ruminants. The present study supports the belief that this “desert-ship” is a fertile immunological subject worthy of in-depth investigation. Some limitations must be addressed in future investigations. First, studies are needed to shed light on the reasons why camels mount a superior innate immune response. Such studies would compare the amount of oxygen consumed by PBL of camels, sheep, goats, and cows, as well as to measure the amount of C3b and iC3b generated during the phagocytic process. In addition, it would be essential to compare the strength of binding of opsonins to their respective receptors and quantify the number of receptors on the phagocytic cells. Finally, it will be of interest to compare antibody responses of these animal species to defined antigens.

## Authors’ Contributions

AA and EHJ: Contributed to conceptualization and design of the study. AA: Performed the laboratory work. EHJ, KA, and EIE: Performed validation. EIE: Performed the statistical analyses. EHJ: Analyzed and interpreted the data. AA: Prepared and wrote the original draft. EHJ, KA, and AA: Reviewed and edited the manuscript. All authors read and approved the final manuscript.
